# Population-specific patterns in assessing molecular subtypes of young black females with triple-negative breast cancer

**DOI:** 10.1038/s41523-025-00731-0

**Published:** 2025-03-11

**Authors:** Padma Sheila Rajagopal, Sonya Reid, Run Fan, Lindsay Venton, Anne Weidner, Mya L. Roberson, Susan Vadaparampil, Xuefeng Wang, Sean Yoder, Marilin Rosa, Melinda Sanders, Paula Gonzalez-Ericsson, Jibril Hirbo, Jennifer G. Whisenant, Jennifer Pietenpol, Fei Ye, Tuya Pal, Brian D. Lehmann

**Affiliations:** 1 Center for Cancer Research, National Cancer Institute, Bethesda, MD USA; 2 Vanderbilt University Medical Center; Department of Medicine, Nashville, TN USA; 3 Vanderbilt University Medical Center; Department of Biostatistics and Bioinformatics, Nashville, TN USA; 4 University of North Carolina; Department of Health Policy and Management, Chapel Hill, NC USA; 5 Moffitt Cancer Center, Tampa, FL USA; 6 Vanderbilt University Medical Center; Department of Biochemistry, Nashville, TN USA

**Keywords:** Breast cancer, Translational research

## Abstract

We determined triple-negative breast cancer (TNBC) subtypes, genetic ancestry, and immune features in a cohort of self-reported Black females with TNBC diagnosed at or below age 50. Among 104 tumors, 34.6% were basal-like 1 (BL1), 17.3% basal-like 2 (BL2), 9.6% luminal androgen receptor (LAR), 26.9% mesenchymal (M), and 11.5% unsubtyped (UNS). Subtypes resembled those seen in Europeans or East Asians, with less LAR (9.6% vs. 14.6–24.4%) and more UNS (11.5% vs. 0–7.5%). “High” proportion of West African ancestry was associated with more LAR (14.9% vs. 4.9%) and less M (25.5% vs. 34.2%). M demonstrated reduced immune activity and was marginally associated with worse overall survival in a multivariate model including stage, West African ancestry, BMI, and TILs, meriting future research. Our study is the largest to date of TNBC subtypes in young Black females. These results reinforce TNBC subtypes’ application across populations and potential use as a prognostic biomarker.

## Introduction

Black females have higher mortality from breast cancer relative to White females in the United States (US) yet remain underrepresented in clinical studies^1^. This higher mortality rate can be attributed in part to overrepresentation of aggressive triple-negative breast cancer (TNBC) in this population^2,3^. TNBC accounts for 10–15% of all breast cancers^4^, yet the relative proportion of TNBCs diagnosed among Black females at or under age 50 in the US is approximately 25–30%^5^. Moreover, West African ancestry is associated with increased rates of TNBC both nationally and internationally^6,7^.

Few targeted treatment options are available for TNBCs compared to other breast cancer subtypes due to the absence of estrogen and progesterone receptors and HER2 amplification. As TNBC is heterogeneous and driven by non-hormonal, non-HER2 molecular mechanisms, molecular subtyping can help identify potential additional treatment targets. Such underlying molecular mechanisms include mesenchymal pathways, upregulation of immune system–related genes or DNA damage repair genes, and activated androgen receptor signaling^8^. Recent advances beyond chemotherapy, such as targeted poly (ADP-ribose) polymerase (PARP) inhibition and immune checkpoint blockade, are shifting the treatment landscape rapidly^9^. However, Black patients continue to be severely underrepresented in landmark clinical trials testing novel experimental therapeutics^10,11^. Adequate representation is critical when considering the generalizability of biomarkers used for prediction and prognostication^12^.

Seminal work by our group to classify TNBCs based on gene expression and identify clinically relevant subtypes was originally published in 2011^13^. TNBCs were initially classified into six molecular subtypes: basal-like 1 (BL1) and basal-like 2 (BL2), immunomodulatory (IM), mesenchymal (M), mesenchymal stem-like (MSL), and luminal androgen receptor type (LAR). This classification was subsequently simplified to four molecular subtypes in 2016: BL1, BL2, M, and LAR^14^. These subtypes have since been applied by multiple national and international groups^15–20^, and shown to predict pathologic complete response to chemotherapy in the early phase setting^21,22^. TNBC subtypes are associated with differential sensitivity to both experimental and standard-of-care therapeutics, including BL1 with DNA-damage repair and cell cycle modulators, BL2 with DNA-damage repair and DNA alkylating agents, LAR with androgen receptor antagonists and PI3K/mTOR pathway inhibitors, and M with kinase inhibitors^15,23,24^.

Through analyses of detailed clinical and molecular data from self-identified Black females with TNBC recruited to a population-based cohort, we determined the distribution of TNBC subtypes in this population relative to other national and international populations. Given that TNBC subtypes, African ancestry (beyond self-reported race), and obesity (via BMI) have known associations with both TILs and overall survival in breast cancer, we also studied the prognostic relationship between these variables using a multivariate model. Our work supports TNBC subtyping as a translational and potentially clinical biomarker for prognostication across populations.

## Results

### Clinical characteristics of study participants

Of 114 participants with primary TNBC in the Black Women: Etiology and Survival of Triple-Negative Breast Cancer (BEST) study, 104 had adequate tumor available for RNA-seq analyses (Supplementary Fig. 1). Clinical characteristics of these participants are summarized in Table 1 (with individual-level data reported in Supplementary Data 1). The median age at diagnosis was 44 years (range 21–50 years old), with the majority (60%) diagnosed with stage II or III disease and a median follow-up time of 10 years. Most samples (93%) were from the primary tumor, with 7% from metastatic sites. Samples from most participants (76.9%) were not treated with chemotherapy prior to tumor tissue collection. However, this reflects solely chemotherapy exposure of the sample (which may be taken from a biopsy or a surgical specimen), versus participants receiving specific neoadjuvant or adjuvant chemotherapy regimens. All but 5 participants received chemotherapy during their treatment.

**Table 1:** Clinical characteristics of analyzed BEST cohort (*N* = 104)

	N	% or Range
Median age/age range	44	21-50
*Died in cohort?*		
Yes	25	24.0%
*Recurrent disease in cohort?*		
Yes	32	30.8%
*Stage*		
I	28	26.9%
II	48	46.2%
III	14	13.5%
IV	5	4.8%
NA	9	8.7%
*Chemotherapy exposure prior to specimen inclusion in BEST cohort*		
No	80	76.9%
Yes	23	22.1%
Unknown	1	0.9%
*West African ancestry*		
High proportion	41	39.4%
Low proportion	47	45.2%
NA	16	14.4%
*Obesity Category (per BMI)*		
Overweight	67	64.4%
Obese	28	26.9%
Normal	8	7.6%
Unknown	1	0.9%

### TNBC subtyping in the BEST cohort relative to other populations

**Fig. 1:**
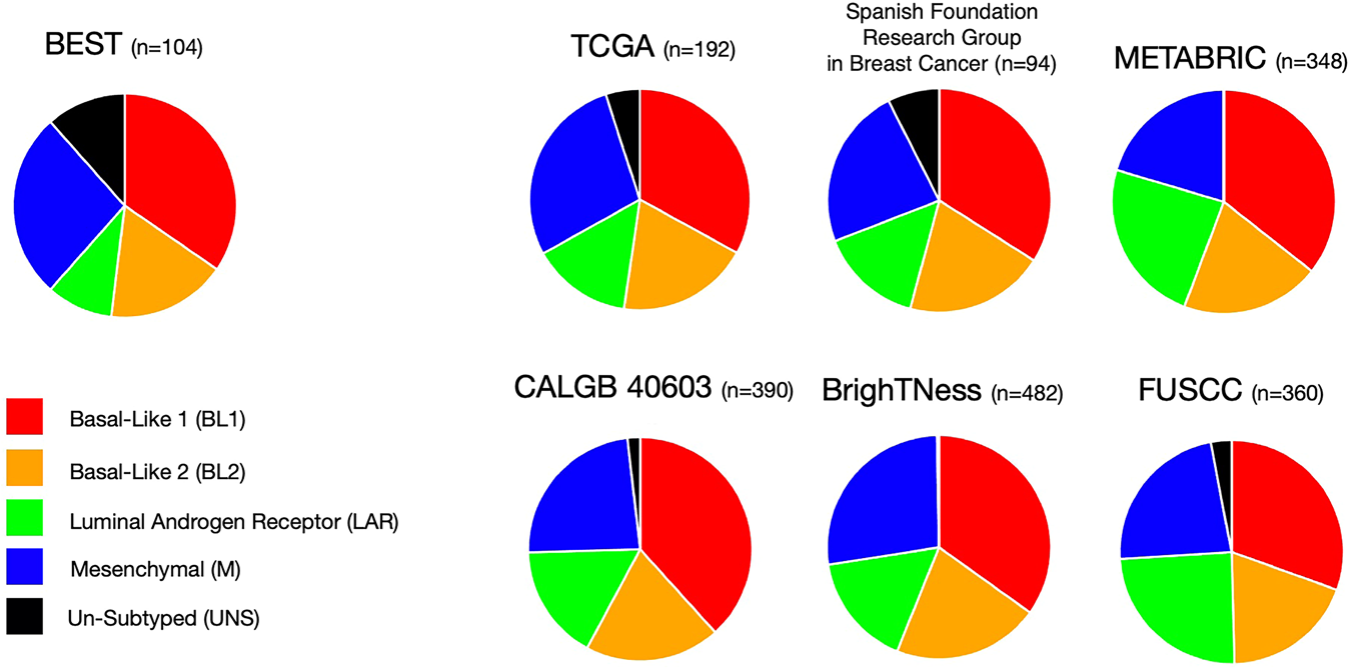
Distribution of TNBC subtypes in the BEST cohort versus other national and international early breast cancer cohorts. The US studies included The Cancer Genome Atlas (TCGA, *n* = 192) and Cancer and Leukemia Group B trial 40603 (CALGB 40603, *n* = 390). European studies included the Spanish Foundation Research Group in Breast Cancer (*n* = 94), the Molecular Taxonomy of Breast Cancer International Consortium (METABRIC, *n* = 348), and the BrighTNess phase III trial of veliparib added to platinum-based neoadjuvant chemotherapy (*n* = 482). The Asian study was performed at Fudan University in China (FUSCC, *n* = 360). Subtype legend on left. Differences between BEST and the other population cohorts were driven largely by the relative proportion of LAR (9.6% in BEST vs. 14.6–24.4% in the other datasets) and untyped tumors (11.5% in BEST compared to 0–7.5% in the other datasets). BL2 differed slightly between BEST and the other cohorts (17.3% in BEST compared to 19.2–21.6%). Proportions of BL1 (30–38.2%) and M (20.4–28.1%) were comparable.

Of the 104 specimens with RNA-seq, 92 were successfully subtyped as follows: 36 with BL1 (34.6%), 18 with BL2 (17.3%), 10 with LAR (9.6%), 28 with M (26.9%) (Fig. 1). The remaining 12 (11.5%) could not be subtyped (“unsubtyped” or UNS) due to low correlation of gene expression levels to a specific subtype. Prior to batch effect correction to adjust for expression variability due to known causes, 65 tumors (62.5%) could not be subtyped (Supplementary Fig. 2). Since the RNA-seq was performed at two different institutions, with different technicians and RNA isolation methods, it was expected that the associated high degree of variability in expression would impact our initial ability to subtype. Final TNBC subtype scores are shown in Supplementary Data 2. Batch effects were observed and corrected for the site of RNA extraction (Moffitt vs. Vanderbilt) and the time between tumor fixation and RNA extraction and RNA-seq. No batch effects were observed by specimen source, sample chemotherapy exposure, PAM50 subtype, or germline pathogenic variant carrier status. Reported TNBC subtypes were determined from batch-corrected, normalized RNA expression data.

Distributions of TNBC subtypes in our study were compared to results from six datasets drawn from the US, Europe, and Asia and showed minor differences in the LAR and BL2 subtypes (Fig. 1). Compared to other TNBC studies (i.e., The Cancer Genome Atlas (TCGA) (US, *n* = 192)^15^, Cancer and Leukemia Group B (CALGB) trial 40603 (US, *n* = 390)^17^, the Spanish Foundation Research Group in Breast Cancer (Spain, *n* = 94)^18^, the Molecular Taxonomy of Breast Cancer International Consortium (METABRIC) (UK, *n* = 348)^15^, the BrighTNess phase III trial of veliparib added to platinum-based neoadjuvant chemotherapy (Europe, *n* = 482)^19^, and Fudan University (FUSCC) (China, *n* = 360)^20^), breast cancers in our BEST study cohort demonstrated a significantly smaller relative proportion of LAR (9.6% vs. 14.6–24.4%; *p* < 0.001) and a significantly higher relative proportion of UNS tumors (11.5% vs. 0–7.5%; *p* < 0.001). BL2 differed slightly between BEST and the other cohorts (17.3% vs. 19.2–21.6%), while proportions of BL1 (34.6% vs. 30–38.2%) and M (26.9% vs. 20.4–28.1%) were comparable.

Comparisons of the TNBC subtyping to PAM50 subtyping showed that most tumors were PAM50 Basal subtype, with more heterogeneity among tumors in the LAR subtype. PAM50 Basal subtypes were seen in only 40% of LAR subtype, and 80% of BL2 subtype, compared to 95–100% for other TNBC subtypes (Supplementary Fig. 3).

### Relationship between genetic ancestry and TNBC subtypes

**Fig. 2:**
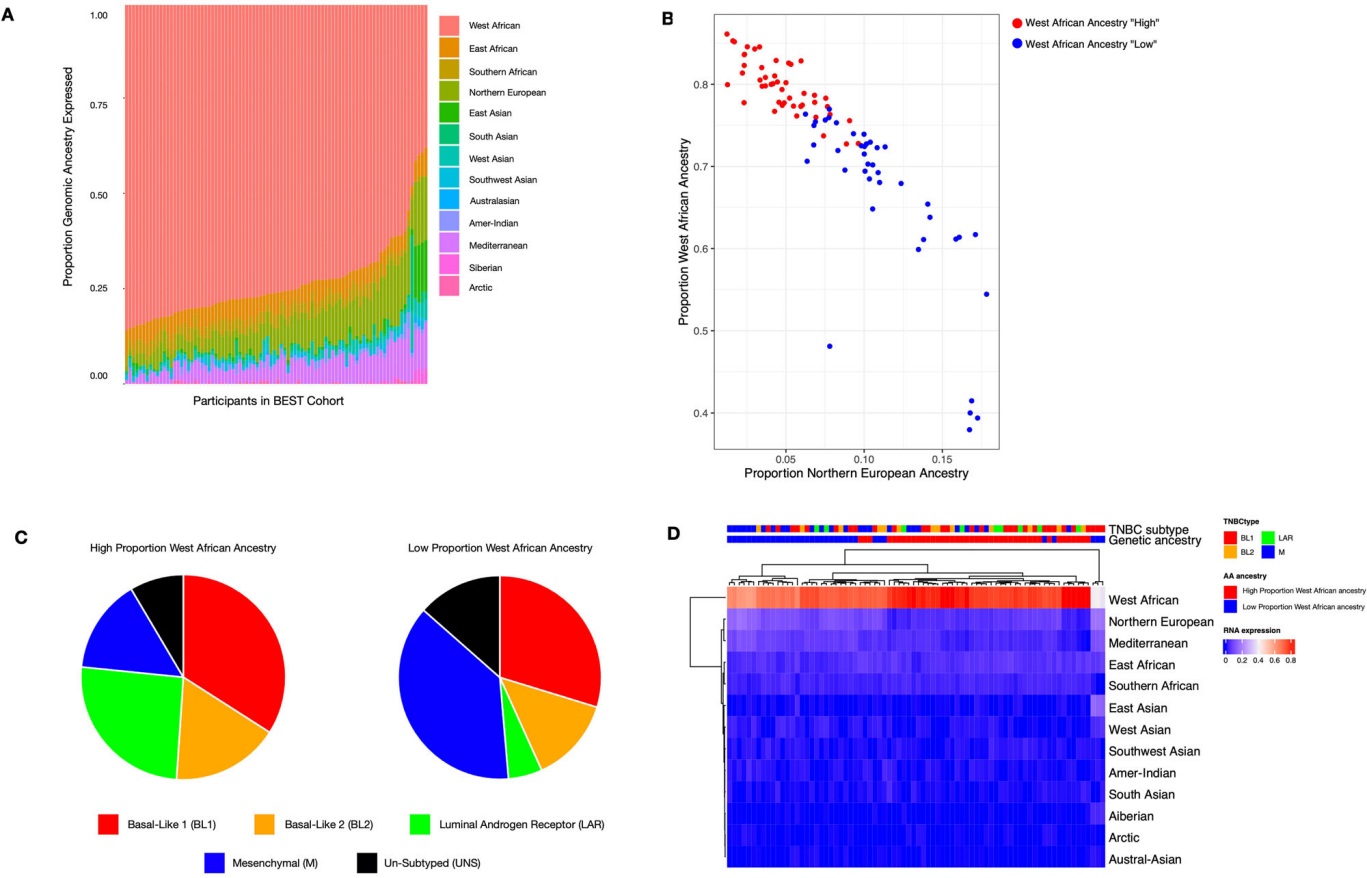
Estimated genetic ancestry distribution in BEST TNBC RNA-seq cohort. Genetic ancestry was estimated from genotypes from multi-locus SNP genotype data with 1000Genomes as the reference. **A** Relative proportion of ancestry estimation per participant with TNBC from the BEST cohort. **B** Distribution of participants with “High” proportional West African ancestry (Red) vs. “Low” relative to European ancestry (Blue). **C** TNBC subtype distribution based on “High” and “low” proportional West African ancestry. **D** Heatmap of unsupervised hierarchical clustering of ancestry proportion in the BEST cohort (“High” proportional West African ancestry in red vs. “Low” in blue), shown with associated TNBC subtypes.

Of the 104 participants, 88 had genotyping-based ancestry data available. The median West African ancestry was 73.4% (range 38–86.1%), and median East African ancestry was 4.8% (range 1.6–7.6%) (Fig. 2A). Phylogenetic clustering of all ancestry proportions revealed two clusters based on percent contribution of West African ancestry: “High” vs. “Low” corresponding to an approximate cutoff threshold of 75% or above (Fig. 2B, Supplementary Fig. 4 for phylogenetic tree diagram).

Distribution of TNBC subtypes in the “High” compared to “Low” ancestry groups differed significantly (*p* = 0.004), driven by the higher relative fraction of LAR subtype (14.9% vs. 4.9%) and lower relative fraction of the M subtype (25.5% vs. 34.2%) in the “High” ancestry group (Fig. 2C, D).

### Relationship between TNBC subtypes and distribution of immune cells in breast tumors

**Fig. 3:**
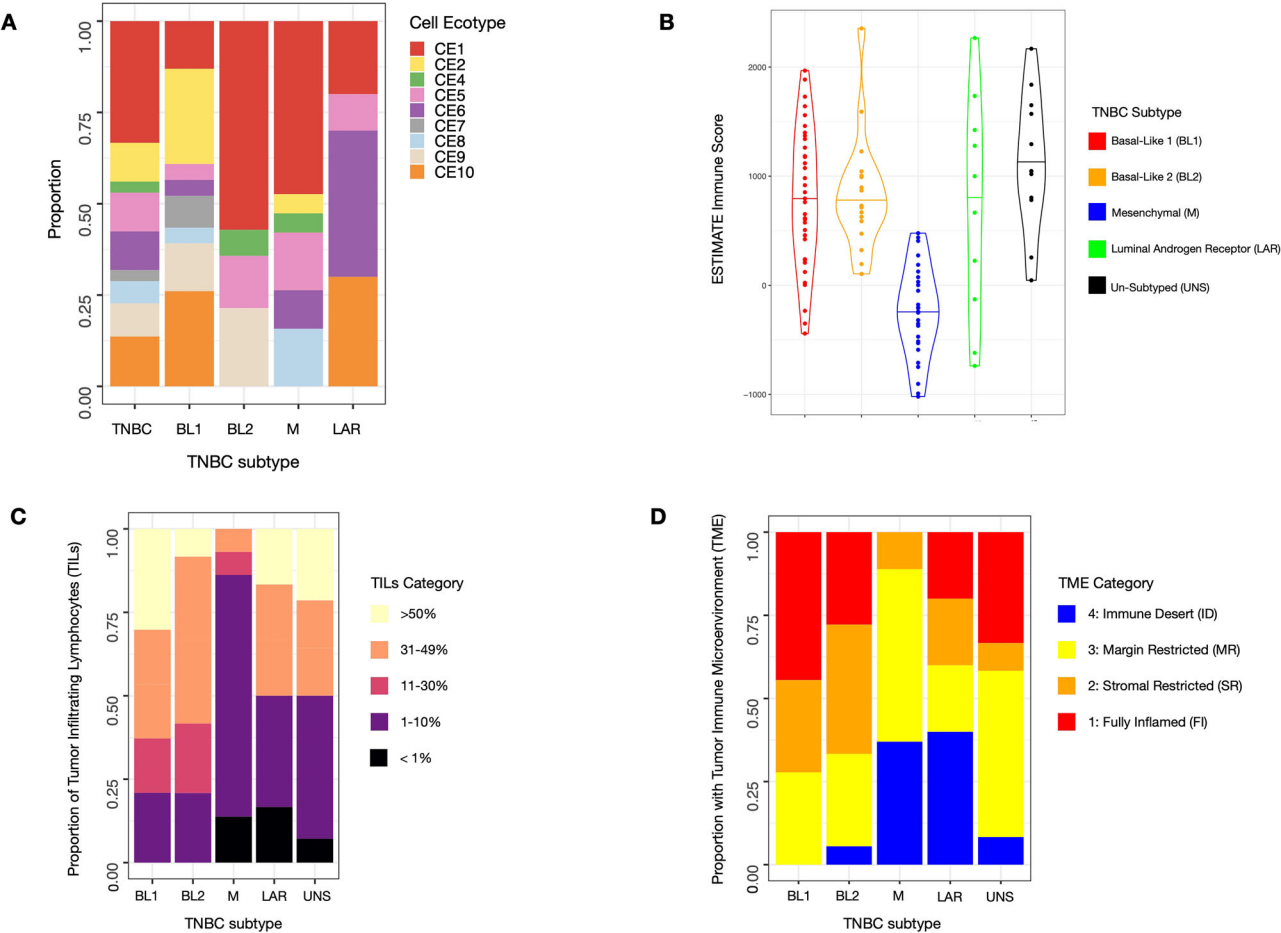
Mesenchymal TNBC tumors are associated with decreased immune cell composition. All measures demonstrated that the M subtype had considerably fewer immune cells than the other subtypes (not controlling for other factors). **A** Relative proportion of cell states across TNBC subtypes using Ecotyper. CE9 and CE10 were the most immunogenic, while CE5 and 8 showed limited immune activity. CE6 reflects immune cell patterns characteristic of normal tissue. **B** Violin plot of ESTIMATE immune scores by TNBC subtype**. C** Proportion of stromal tumor-infiltrating lymphocytes (TILs) per participant sample across TNBC subtypes. Categories are based on percent TIL distributions. **D** Tumor immune microenvironment per participant samples across TNBC subtype. Immune desert and margin restricted were the least immune activated, and stroma restricted or fully inflamed were the most.

RNA-seq data was used to infer immune cell states and content with Ecotyper^25^ and ESTIMATE^26^. Through Ecotyper, statistically significant associations were identified between carcinoma ecotypes (CEs) (defined through transcriptional cell “states” and inferred cell types) and TNBC subtypes (Fig. 3A, Supplementary Fig. 5 for detailed heatmap). BL1 tumors were associated with CE9 and CE10, which were the most immunogenic ecotypes. CE9 included activated B-cells, proinflammatory epithelia, NK cells, and exhausted CD4/CD8 T-cells, while CE10 included naïve B cells, naïve CD4/CD8 T-cells, mast cells, and monocytes. BL2 was associated with CE2, characterized by more basal-like, proliferative cell states. LAR was associated with CE6, characterized by immune cell patterns typical of normal tissue. M was associated with CE5 and CE8, with plasma cells and limited immune activity, and had significantly fewer immune cells inferred compared to other subtypes in ESTIMATE (median score −225 vs. 828, *p* < 0.001 by two-tailed *T* test) (Fig. 3B).

H&E slide pathology review for stromal tumor-infiltrating lymphocytes (TILs) and tumor-immune microenvironments (TMEs) confirmed fewer stromal TILs in the M subtype (Fig. 3C) and a lower proportion of fully inflamed or stromal-restricted TMEs (Fig. 3D), consistent with Ecotyper and ESTIMATE results. Supplementary Data 3 reviews actual immune calls at the individual participant level.

### Relationship between TNBC subtypes and BMI

Differences in TNBC subtype distributions across BMI categories did not reach statistical significance (*p* = 0.07). However, only 8 participants were within a “normal” BMI range (Supplementary Data 1). Obese participants were more likely than overweight participants to have M subtype tumors (34.3% versus 10.7%). There were no LAR tumors among participants with a “normal” BMI.

### Contribution of TNBC subtype, African ancestry, immune features, and BMI to overall survival

**Fig. 4:**
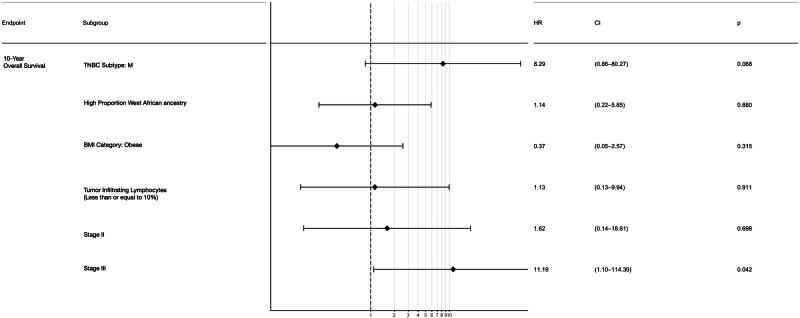
Forest plot displays initial multivariate Cox proportional 10-year overall survival hazard ratios for TNBC subtype, West African ancestry, BMI, TILs, and tumor stage. Sixty-three participants, none of whose samples were exposed to chemotherapy, had data in all categories with events for analysis. Six deaths were observed among these participants. Tumor microenvironment (TME) was not included in this model as this was collinear with stromal tumor-infiltrating lymphocytes (TILs). The reference categories for the initial multivariate model were as follows: “Other” TNBC subtypes, “Low” proportional West African ancestry, not obese, stromal TILs < or equal to 10%, and stage 1 disease. The median follow-up time was 10 years; participants were censored to time of last follow-up. Backwards selection was used to retain the final variables in the model, which were TNBC subtype and disease stage.

TNBC subtypes, along with West African ancestry, TILs, BMI, and stage, were evaluated for association with 10-year overall survival (Fig. 4). After limiting our analysis to specimens that were not previously exposed to chemotherapy, our dataset included 63 participants with data available in all categories (excluding stage 4 cases), of whom 6 died during the follow-up period.

The final variables retained in the model after backwards selection were M subtype (relative to other TNBC subtypes, HR: 8.29, 95% CI 0.86–80.27, *p* = 0.06) and disease stage (Stage III disease HR: 11.19, 95% CI: 1.10–114.30, *p* = 0.04). “High” proportional West African ancestry, BMI (obesity), and stromal TILs were not retained after backwards selection. TME was collinear with stromal TILs and therefore not included in the initial model. Categories were collapsed where necessary to improve statistical power (see “Methods”). Partial effects plots for the final model for survival are shown in Supplementary Fig. 6.

## Discussion

This study represents the largest analysis to date of TNBC subtypes in self-identified Black females with TNBC, reinforcing TNBC subtypes as a potential biomarker for prognostication in TNBC. While proportions of BL1 and M subtypes were similar compared to European or Asian cohorts (with a slight increase in proportions of BL2), there were smaller relative proportions of LAR subtypes and larger proportions of UNS tumors, where these tumors showed low correlations of expression to all subtypes. Overall, the LAR subtype was seen more commonly in those with a higher proportion of West African ancestry, while the M subtype was seen more commonly in those with proportionally lower contribution. In evaluating immune features, we also found that the M subtype was associated with fewer stromal TILs and a lower proportion of fully inflamed or stromal-restricted TMEs, consistent with prior analyses in predominantly European ancestry TNBC patients from the US (TCGA)^15^ and Europe (METABRIC)^15^.

Significant differences in TNBC subtypes by proportion of West African ancestry were observed, with more M subtype tumors and fewer LAR subtype tumors among participants with a lower relative proportion of West African ancestry. These findings related to ancestry are consistent with the single other prior study among females of African ancestry with breast cancer^16^. In that study of nine African Americans, when compared to six Ghanaians, M subtype cancers were overrepresented among African Americans, who had lower proportional West African ancestry (3 out of 9 cancers vs. 1 out of 6)^16^. Given prior studies showing differential utility of genomics tools with race-specific differences in predictive accuracy, as seen for Oncotype (where ancestry was not characterized)^27^, our results are critical to demonstrating how TNBC subtyping may be applied among individuals of African ancestry.

These differences in TNBC subtype distributions, as identified in our study, may be related to our cohort’s focus on females diagnosed with invasive breast cancer at or below age 50, particularly the lower proportion of the LAR subtype observed compared to other studies. The BEST cohort is specifically intended to capture cancers in women at or below age 50. Prior studies have shown the LAR subtype to be associated with worse survival^14,15^. This was previously noted in the context of tumor heterogeneity based on PAM50 subtyping, which we also observed^23^. In contrast to the young age at diagnosis in our study (median age 44), the median age in other reported studies among TNBC patients is older, including TCGA (median age 53), METABRIC (median age 53.9)^15,28,29^, Spanish cohort (median age 51)^18^, and the BrighTNess and the FUSCC cohort (over half of the participants were over age 50)^30,31^. Further research is required to determine the extent to which menopausal status (and associated fluctuations in estrogen, progesterone, and testosterone) may be a mediator of the differences we observed in the BEST cohort with regard to the LAR subtype and overall survival. Given that participants in this cohort were ages 50 or below, most had not undergone menopause, and we were therefore unable to assess this in our study. The nuances observed in our study about age, the LAR subtype, and overall survival (relative to prior studies) is a translationally essential highlight noted in this work.

M subtype was marginally associated with worse overall survival after accounting for ancestry, BMI, TILs, and stage, but did not reach statistical significance and had wide confidence intervals given the number of participants (*n* = 63) and deaths (*n* = 6). Encouragingly, this model recapitulates previously observed patterns. The association between M subtype and lower measures of immunogenicity (based on TILs and TME) is consistent with prior studies^15,21^. Interestingly, stromal TILs as a variable was removed in backwards selection and did not show as significant an association relative to M subtype. These findings encourage continued research into TNBC subtyping as a prognostic biomarker when accounting for other features.

Interrelationships between TILs, ancestry, and BMI as related to breast cancer outcomes have been previously reported, but it remains challenging to tease apart the relative contributions of these factors^6,32,33^. TILs or TME features have been associated in the literature with improved overall survival in TNBC, but largely in datasets that were not diverse^34–37^. African ancestry has also been shown to be associated with an increased presence of immune cells in TNBCs, with these immune cells not necessarily having the same beneficial clinical implications^16,32,38–43^. Given that the median proportion of West African ancestry among participants in the BEST cohort was 0.75, we may observe clearer association with prognostication in a more ancestrally heterogeneous population. In primary TNBCs, obesity is associated with mixed molecular patterns related to chronic inflammation, including both increased and paradoxically suppressed immune features^44,45^. Patients may also experience worse outcomes^46^. Accordingly, obesity has been reported to have an interaction effect with TILs that modifies their prognostic interpretation in breast cancer^47–49^. The finding of an increased proportion of obese patients with M subtype in our study highlights this challenge of relating obesity to consistent immune patterns in breast cancer. Given the complexity in deciphering the relative importance of these confounding clinical features, our findings, while meriting further research, reinforce the necessity of clinically applicable prognostic biomarkers that remain so even after accounting for ancestry and BMI.

The current study has many strengths, including the integrated clinical and molecular data needed to capture the nuances of overlapping clinical features in individuals of African ancestry, robust molecular and pathological annotation of immune features of tumors to triangulate findings, and population-based design to enhance generalizability of results inclusive of participants treated across academic and community sites. Furthermore, we were able to assess batch effect correction, which demonstrated the importance of including location of RNA extraction and time from diagnosis to extraction or sequencing in sample collection to reduce the number of unassigned samples.

Despite these strengths, this work has some limitations. We have acknowledged that our multivariate model, while of interest, has wide confidence intervals and does not reach statistical significance given the low number of events (6 deaths among 63 analyzed participants). We are also not able to provide insight into potential associations with treatment responses. We do not have specific chemotherapy regimen information or specific details (such as timing) of radiation treatment. Additionally, given when patients were recruited, immunotherapy was not yet part of standard of care. Further research is needed to validate our findings in the context of current regimens.

This study also included a limited number of participants with metastatic disease. De novo metastatic breast cancer represents 3–6% of all breast cancers diagnosed in the US (consistent with the 5/104 participants in our study)^50^. Despite our sample being representative of de novo metastatic disease, we were not powered to capture TNBC subtypes in the metastatic setting through sampling alone. Additionally, serial tissue biopsies in metastatic disease were not as routinely captured as part of standard-of-care breast cancer treatment during the time period when participants were recruited to this study. TNBC subtypes may change in response to treatment, which may eventually guide treatment strategies in the context of significant residual disease^51^, suggesting the importance of serial analyses on tumor specimens in future efforts.

We observed a larger proportion of UNS tumors compared to other cohorts, even after batch effect correction based on site and time from preservation to sequencing. The tumors in this study met initial RNA-seq quality controls. We observed that nine of the 12 UNS tumors had a strong correlation to the mesenchymal subtype, which is reflective of adjacent stromal tissue. This suggests that the UNS tumors we observed may reflect potential poor tumor cellularity rather than a biological phenomenon.

With regard to ancestral populations, we sought to use a resource that aggregated multiple publicly available genotyping datasets. However, any reliance on population groupings derived through common HapMap SNPs (which were predominantly ascertained from European ancestral populations) and the current genome reference may potentially be blunted regarding admixture in non-European populations. Ideally, implementation of the human pangenome as a reference may allow this analysis and many such others to be improved upon.

This study has two points of overall significance. First, as the largest study to date to evaluate TNBC subtypes and associated molecular and clinical data in young, self-identified Black females in the US with invasive breast cancer, our findings support the comparability of TNBC subtyping in tumors from patients of African ancestry in those from other populations. Second, findings from our study suggest the value of TNBC subtyping as a potential prognostic biomarker after accounting for ancestry and BMI. Many prognostic studies of immune features in breast cancer do not include these conflating clinical factors. Better characterization of the true prognostic role of biomarkers capturing immune phenomena is particularly important for individuals of African ancestry, who experience active disparities in access to trials and treatment, but will be critical for serving all patients^52^.

## Methods

### BEST study participants/cohort methods

The BEST study is an actively ongoing cohort study of self-identified Black females diagnosed with invasive breast cancer at or below the age of 50 between 2005 and 2016 recruited through the state cancer registries in Florida or Tennessee. This cohort was established to study factors contributing to the epidemiologic disparities in incidence of TNBC and outcomes among young Black women, including germline susceptibility mutations (over-represented among early onset/pre-menopausal breast cancers). Germline DNA, tumor RNA (both whole-transcriptome RNA-seq and Nanostring PAM50), and clinical data were collected. Data abstracted from medical records was supplemented with data from state cancer registries and self-reported questionnaires. Self-reported questionnaires focused on socio-demographic, epidemiologic, and lifestyle factors. 10-year survival outcomes were collected from medical records, the TransUnion VitalChek database, and follow-up data from the Florida and Tennessee state cancer registries. This study has been reviewed and approved by the Institutional Review Board at Vanderbilt-Ingram Cancer Center (IRB #170233). This study has also been reviewed and approved by the Florida Department of Health (Study Number: 2011-05-VBU) and the Tennessee Department of Health (Study Number: TDHIRB-2019-0139). This study complies with all relevant ethical regulations regarding patient data, in line with ethical norms and standards in the Declaration of Helsinki.

Eligibility for inclusion in this specific analysis was based on reported immunohistochemistry (IHC) determination of hormone receptor (less than 1%) and HER2 status (negative as determined by clinical team), as well as availability of RNA-seq of sufficient quality to undergo TNBCtype-4 subtyping. Medical records and pathology reports were abstracted and supplemented with cancer registry and self-reported questionnaire data to obtain estrogen receptor, progesterone receptor, and HER2 receptor status.

### RNA-seq from banked tumor samples

Participants’ formalin-fixed paraffin-embedded (FFPE) tumor tissue blocks or unstained FFPE slides were banked at the Moffitt Comprehensive Cancer Center and Vanderbilt-Ingram Cancer Center with paired hematoxylin and eosin (H&E) slides between 2005 and 2017. H&E slides were scanned and manually evaluated to annotate tumor area as a guide for dissection. The Translational Pathology Shared Resource at Vanderbilt macro-dissected the tissue to enrich for tumor cells based on these annotations. RNA was stored at -80C and extracted from the FFPE tumor tissue blocks between 2016 and 2022. Samples were extracted at Vanderbilt using the Covaris RNA FFPE kit and at Moffitt using the Ambion RecoverAll Total Nucleic Acid Isolation Kit. Between 2022 and 2023, whole transcriptome RNA-sequencing (RNA-seq) on extracted tumor RNA was performed using the Vanderbilt VANTAGE platform, which uses paired-end 150 bp on the Illumina NovaSeq 6000 and targets an average of 50 M reads per sample. Library preparation at Vanderbilt was performed via Ribo-Zero Plus rRNA Depletion.

Extracted tumor RNA was also submitted to the Nanostring nCounter platform, as well as the commercial Prosigna assay for PAM50 subtyping. NanoString nCounter capture and reporter probes for the PAM50 and Panel-Plus CodeSets targeting 20 additional genes were processed according to the manufacturer’s protocol (NanoString Technologies, Seattle, WA). Briefly, the probes were hybridized at 65°C for 16 hours to FFPE-extracted RNA using an adjusted input amount of 50 ng or greater depending on the DV300 value reported from an Agilent TapeStation RNA ScreenTape (Agilent Technologies, Santa Clara, CA). Washing and cartridge immobilization was performed on the nCounter PrepStation using the high-sensitivity mode according to the manufacturer’s protocol. The cartridge was scanned at 555 fields of view (FOV) on the nCounter Digital Analyzer, and the data were reviewed for quality using the NanoString nSolver Analysis Software v4.0.

### Sequencing data generation and batch effect correction

FASTQ files of RNA-seq were processed with alignment to Hg38 using STAR aligner 2-pass and standardized quality control measures (Fast QC, PicardTools). Gene-level read counts were quantified using subREAD. Count level gene expression data was corrected for batch effects related to known variables, including time from fixation and metastatic tissue with the ComBat_seq function (sva v3.35.2). Count level data were corrected for batch effects from the extraction batch (limma v 3.56.2), adjusted count data were normalized, and differentially expressed genes were identified using DESeq2 (v1.30.1), correcting for the extraction method. Sources of potential batch effect included batch number, time to RNA extraction and sequencing, geographic location of RNA extraction, specimen source (primary vs. metastatic tumor site), sample exposure to chemotherapy, PAM50 subtype, and germline carrier status.

### TNBC subtype assessment

After normalization and visualization with principal components analysis, batch effect correction was applied to account for (1) the site of RNA extraction (Moffitt vs. Vanderbilt), (2) the time between tumor fixation and RNA extraction, and (3) the time from RNA extraction to RNA-seq (Supplementary Fig. 2).

Normalized, batch-corrected, log2-transformed RNA expression values were used to determine TNBCtype (http://cbc.mc.vanderbilt.edu/tnbc/) as previously described^14,53^. The highest correlation coefficients were used to assign BL1, BL2, M or LAR.

### Genetic ancestry of participants

Saliva samples were collected using an Oragene Self-Collection kit (DNA Genotek, Inc.) and shipped to the investigators for DNA extraction. NanoDrop and Aubit technologies were used for DNA quantification and quality assessment. DNA samples were stored at -80°C prior to genotyping. Samples were genotyped using OncoArray or the Multi-Ethnic Global Array (MEGA). Standard sample- and variant-level quality control procedures were performed. Ancestry proportions for each individual were estimated from multi-locus SNP genotype data using the maximum likelihood-based ADMIXTURE method (39 SNPs), and assigned ancestral population percent contributions using the Dodecad Ancestry Project’s “globe13” calculator (“West African,” “East African,” “Paleo-African” (which we revised for clarity in this paper as “Southern African”), “Northern European,” “East Asian,” “South Asian,” “West Asian,” “Southwest Asian,” “Austral-Asian,” “Amer-Indian,” “Mediterranean,” “Siberian,” and “Arctic”) (https://dodecad.blogspot.com/2012/10/globe13-calculator.html). Population groupings from this project were used in order to leverage its inclusion of over 25 publicly available datasets, including 1000 Genomes and the Human Genome Diversity Project. R package radmixture was used to estimate genetic ancestry proportions. West African ancestry “High” vs. “Low” categorization was determined using phylogenetic proximity, with an approximate cutoff threshold of African ancestry contribution of greater than or equal to 75%.

### Immune cell characterization

Relative proportions of immune cells in breast tumors were inferred from bulk RNA-seq gene expression data via Ecotyper, which estimates immune cell states, and ESTIMATE, which uses gene expression to infer the fraction of stromal and immune cells in tumors^25,26^. Pathological assessment was performed to validate and build on RNA-based immune cell characterizations, with scoring of stromal TILs and evaluation of tumor-immune microenvironments (TMEs) by four trained pathologists manually reviewing either physical or scanned H&E slides and blinded to the clinical and experimental data. TILs were scored according to the TIL-WG guidelines stratified as follows: 0; <1%, 1; 1 to 10%, 2; 11–30%, 3; 31–49%, and 4; >50%^54,55^. Characterization of the TME was binned into 4 categories as previously described^56^. No accumulation of lymphocytes in either stroma or tumor was classified as immune desert (ID). Accumulation of lymphocytes at the tumor periphery (>25% of circumference) and ≤10% in the tumor stroma were classified as margin-restricted (MR). Tumors with predominantly stromal infiltration of lymphocytes were classified as stroma-restricted (SR). Tumors with epithelial and stromal infiltration of lymphocytes with infiltration above the median were classified as fully inflamed (FI).

### Statistical analyses

Categorical variables were summarized using frequencies and percentages, while continuous variables were summarized by mean, medians, and ranges. Statistical significance in comparing differences across TNBC subtypes was assessed via Chi-squared test. For Ecotyper data, statistical significance was determined by identifying a mean of Ecotyper correlation values for all samples within a given subtype, then performing a standard two-tailed *T* test against all other subtypes and adjusting for multiple testing via Bonferroni correction (*p* < 0.01).

Cox proportional hazards and the log-rank test were used for survival analysis. Categories were collapsed as needed to improve statistical power. TNBC subtypes were analyzed from the baseline of BL1, but this was condensed to M subtype vs. “Other” TNBC subtypes in the final model. Stromal TILs and TME were analyzed in their respective categories and further binary categories of less than or equal to vs. greater than 10% stromal TILs and immune restricted vs. inflamed. West African ancestry was binarized to proportionally “High” versus “Low” ancestry as described. BMI was categorized as three categories: normal, overweight, or obese. This was binarized to obese vs. not obese in the multivariate model, with “not obese” primarily including overweight participants. Only participants with stage I, II, III disease were included. The reference categories for the multivariate model were as follows: “Other” TNBC subtypes, stromal TILs < or equal to 10%, “Low” proportional West African ancestry, not obese, and stage 1 disease. Statistical analyses were performed and figures generated in R v.4.3.3 and Microsoft Excel v.16.83.

## Supplementary information

Supplementary Figures and Data Legends

Supplementary Data

## Data Availability

Deidentified genomic and transcriptomic data will be made available through dbGaP and GEO upon publication.

## Code availability

The code for TNBC subtyping has already been made publicly available in prior manuscripts. No novel code was generated in this manuscript.
