# Supplementary material for: Population-specific patterns in assessing molecular subtypes of young black females with triple-negative breast cancer
Source: NPJ Breast Cancer. 2025 Mar 11;11:28. doi: 10.1038/s41523-025-00731-0 (PMC11897140; doi:10.1038/s41523-025-00731-0)
Supplement: Supplementary file 1 — Supplementary Figures and Data Legends [file 41523_2025_731_MOESM1_ESM.docx]

**Supplementary Figures**

**
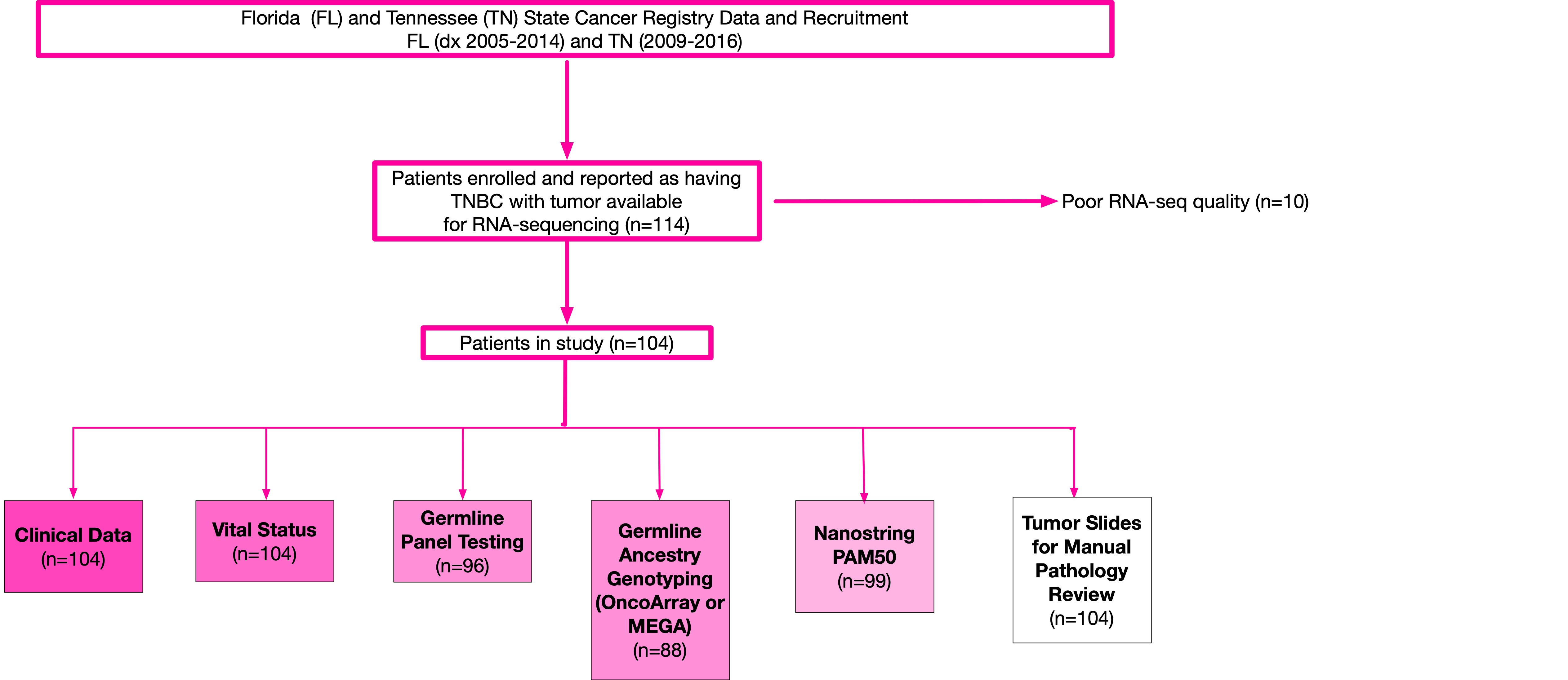
Supplementary Figure 1: Participants in the BEST cohort and data types available from participants included in this analysis.** Data abstracted from medical records were supplemented with data from state cancer registries and self-reported questionnaires. 10-year survival outcomes were collected from medical records, the TransUnion VitalChek database, and follow-up data from the Florida and Tennessee state cancer registries. Participants were excluded if RNA-seq was not adequate.

**
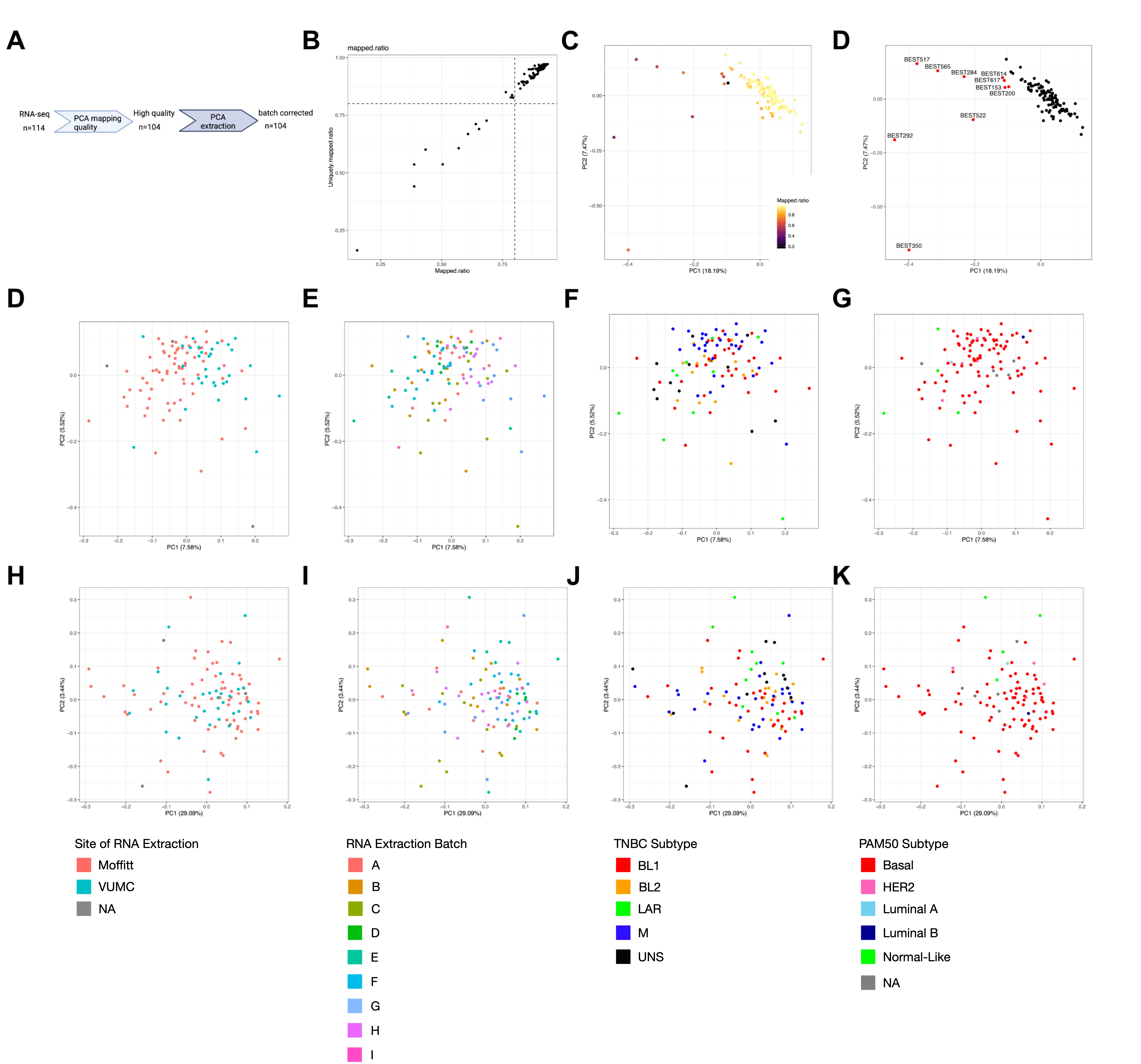
**

**Supplementary Figure 2: RNA-seq data processing and batch correction. A.** Workflow of RNA-seq quality control and batch correction. **B.** RNA-seq plot of mapping ratios. **C.** PCA of mapping quality. Batch correction by site of RNA extraction is shown in panels **D** (before) and **H** (after), with legend of sites (Moffitt, Vanderbilt or unknown). Effect of correction by actual batch of RNA extraction is shown in panels **E** (before) and **I** (after), with the legend of batches A-I below**.** Batch correction by TNBC subtype is shown in panels **F** (before) and **J** (after) with legend below**.** Batch correction by PAM50 subtype is shown in panels **G** (before) and **K** (after) with legend below. Batch effect correction ultimately was applied to account for site of RNA extraction (as well as time between tumor fixation and RNA extraction and time from RNA extraction to sequencing).

**
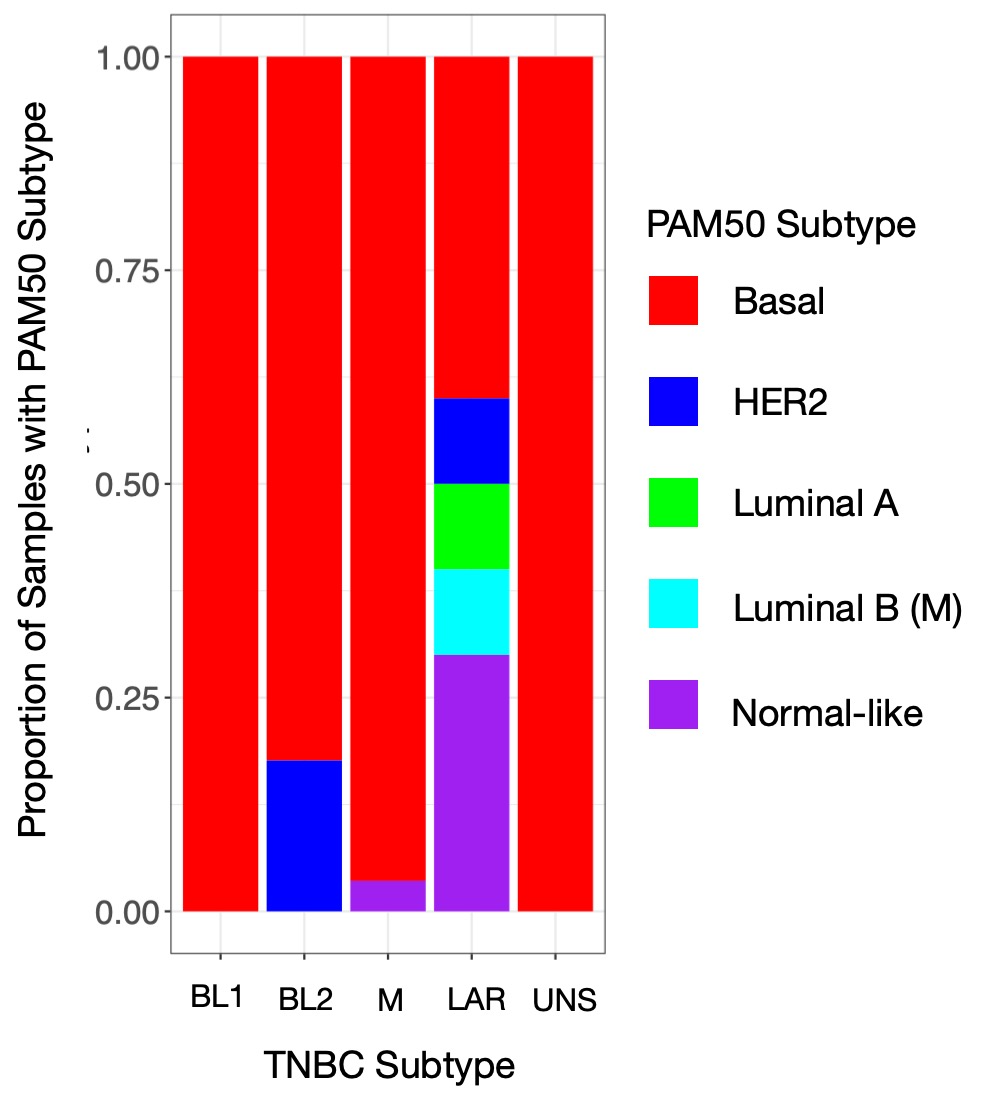
**

**Supplementary Figure 3: PAM50 and *ESR1* expression analysis of TNBC subtypes. A.** Distribution of PAM50 subtype scores by TNBC subtypes. PAM50 subtypes were predominantly Basal across TNBC subtypes, except for the LAR subtype, which showed the most non-Basal PAM50 subtyping. **B.** Violin plot of ESR1 expression as measured by TNBC subtype to evaluate if the LAR subtype heterogeneity in PAM50 subtype was potentially mediated by estrogen, given that tumors were included based on immunohistochemistry.

**
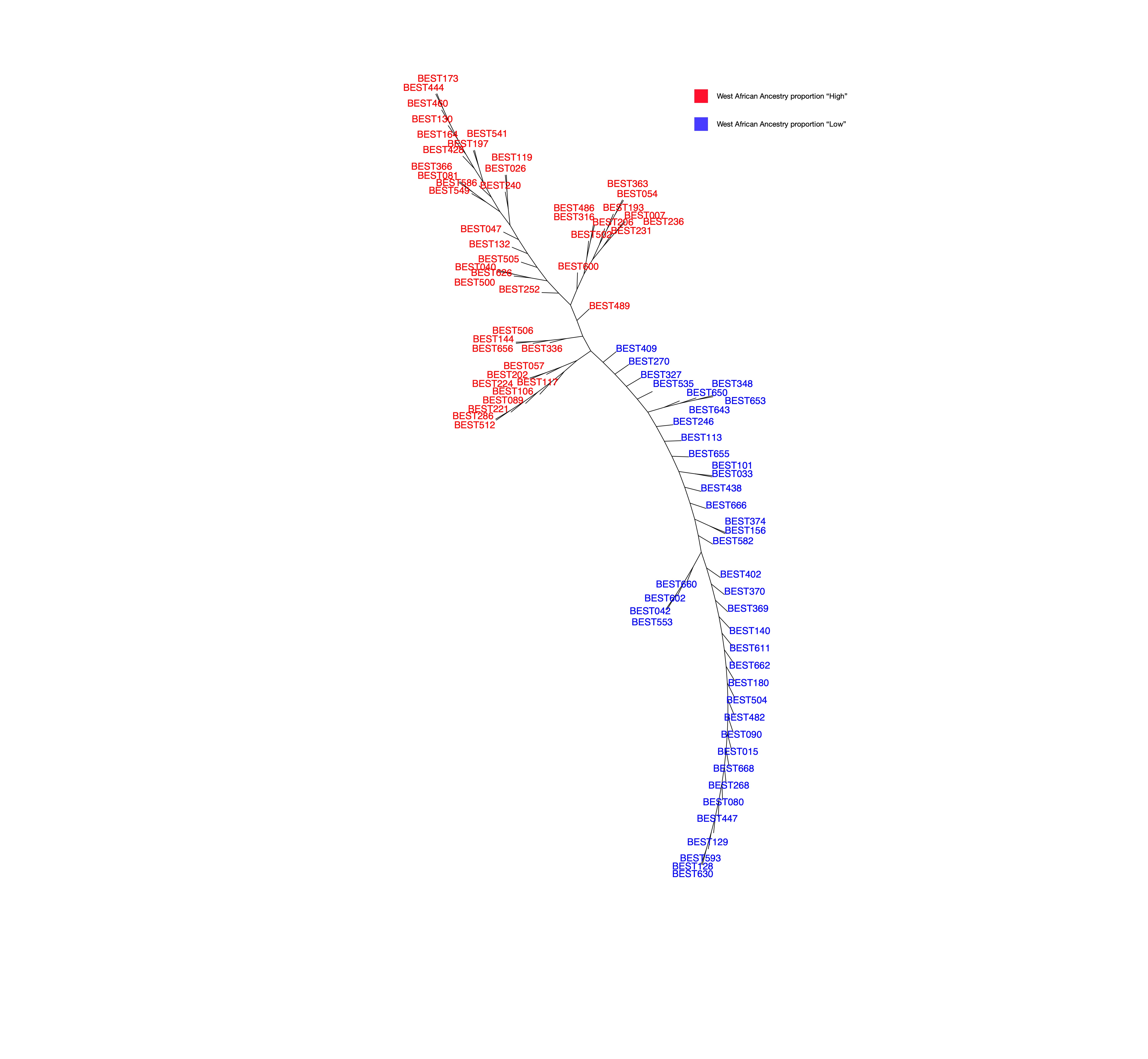
Supplementary Figure 4: Phylogenetic clustering of ancestry proportions identifies two distinct populations.** Phylogenetic clustering of BEST cohort based on germline genotyping SNPs, used to classify patients as West African ancestry proportionally “High” (in red) vs. “Low” (in blue). Clustering bifurcation corresponded to an approximate cutoff threshold of 75% or above for “High.”

**
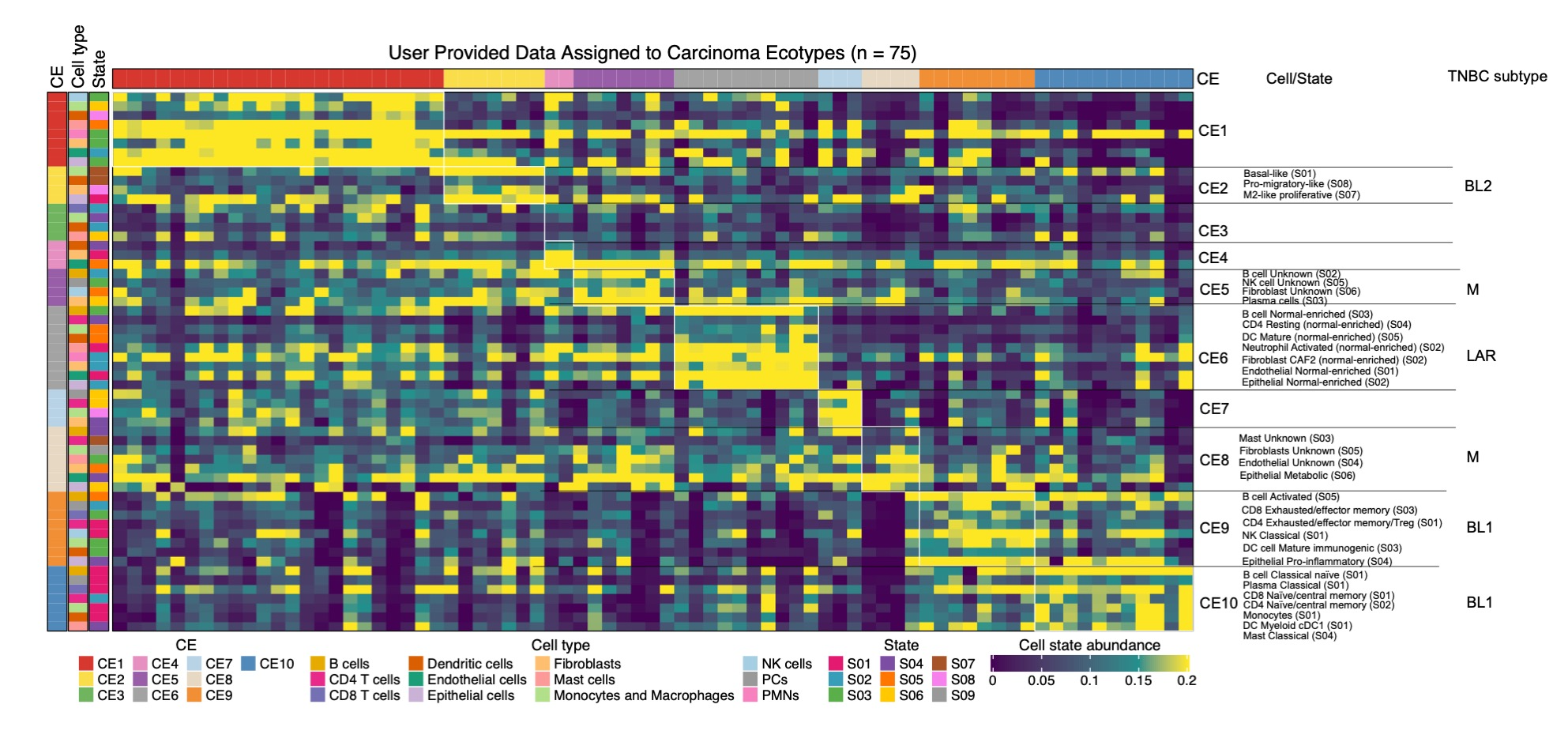
**

**Supplementary Figure 5: Identification of cell ecotypes and states** Heatmap generated from Ecotyper. Cell states (“States”) are defined in Ecotyper as “cell-type-specific transcriptional programs.” Cell ecotypes (“CEs”) are defined in Ecotyper as “multicellular communities.” The heat map shows the distribution of cell states, inferred types associated with each ecotype, and the ecotypes associated with each TNBC subtype.


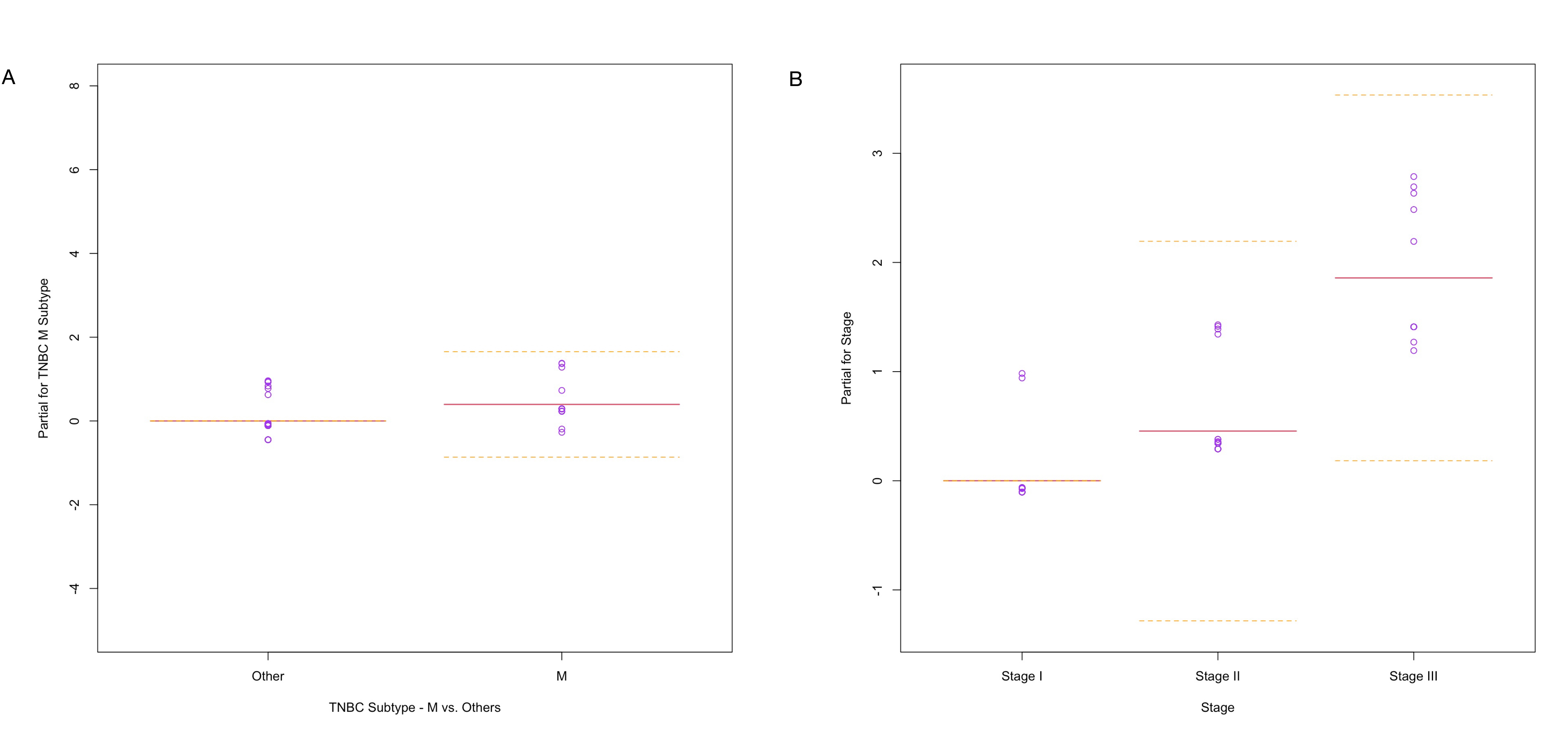


**Supplementary Figure 6: Partial effects plots for survival analysis.** Partial effects plots that demonstrate extent to which individual regression terms contribute to the survival outcome in the final model. Partial effects plots display variable (x-axis) and partial residuals (y-axis) for (A) TNBC subtype and (B) disease stage. Variables set at 0 were used as the baseline for the model. Purple dots represent individual residual points. These plots are in lieu of Kaplan-Meier curves.

**Supplementary Data**

**Supplementary Data 1: Individual-Level Data of Analyzed BEST Cohort Participants.** Clinical and translational data described in the study per participant.

**Supplementary Data 2: TNBC subtype scores and assignments after batch correction.** TNBC subtypes reported per participant with calculations.

**Supplementary Data 3: Manual immune scoring of participant tumors.** % stromal tumor infiltrating lymphocytes (TILs) from 0 (<1%) to 4 (>50%) and tumor immune microenvironment (TME) classifications from 1 (immune desert) to 4 (fully inflamed). Scoring performed as per Grousso et al (51).
